# MicroRNA-16 inhibits feto-maternal angiogenesis and causes recurrent spontaneous abortion by targeting vascular endothelial growth factor

**DOI:** 10.1038/srep35536

**Published:** 2016-10-17

**Authors:** Yongsheng Zhu, Hong Lu, Zhenghao Huo, Zhanbin Ma, Jie Dang, Wei Dang, Lin Pan, Jing Chen, Huijun Zhong

**Affiliations:** 1 Xi’an Jiao Tong University, College of Forensic Science, Xi’an, Shaanxi, PR China; 2 Ningxia Medical University, Key Laboratory of Fertility Preservation and Maintenance of Ministry of Education, Key Laboratory of Reproduction and Genetics, Department of Medical Genetic and Cell Biology, Yinchuan, Ningxia, PR China; 3 Xi’an Mental Health Center, The Sixth Ward, Xi’an, Shaanxi, PR China; 4 General Hospital of Ningxia Medical University, Department of Clinical Laboratory, Yinchuan, Ningxia, PR China

## Abstract

Recurrent spontaneous abortion (RSA) is a common health problem that affects women of reproductive age. Recent studies have indicated that microRNAs are important factors in miscarriage. This study investigated the role of miR-16 in regulating vascular endothelial growth factor (VEGF) expression and the pathogenesis of RSA. In this report, clinical samples revealed that miR-16 expression was significantly elevated in the villi and decidua of RSA patients. *In vitro*, miR-16 upregulation inhibited human umbilical vein endothelial cell proliferation, migration and tube formation. Conversely, the downregulation of miR-16 reversed these effects. *In vivo*, we demonstrated that abnormal miR-16 levels affect the weights of the placenta and embryo and the number of progeny and microvascular density, as well as cause recurrent abortions by controlling VEGF expression in pregnant mice. VEGF, a potential target gene of miR-16, was inversely correlated with miR-16 expression in the decidua of clinical samples. Furthermore, the luciferase reporter system demonstrated that miR-16 was found to directly downregulate the expression of VEGF by binding a specific sequence of its 3′-untranslated region (3′UTR). Collectively, these data strongly suggest that miR-16 regulates placental angiogenesis and development by targeting VEGF expression and is involved in the pathogenesis of RSA.

Spontaneous abortion, a common complication of pregnancy, accounts for approximately 15% of clinically recognized pregnancies^1^. Recurrent spontaneous abortion (RSA) has been defined as the unintentional termination of two or more consecutive pregnancies before 20 weeks of gestation^2^. The World Health Organization (WHO) has estimated that 1–5% of all women suffer from recurrent pregnancy loss during reproductive age^3^. The risk of a spontaneous abortion was 8.9% in women aged 20–24 years and 74.7% in those aged 45 years or older^4^. The causes of RSA have been generally recognized to include anatomical issues, genetics and hormonal abnormalities, exposure to environmental factors, infection, smoking and alcohol consumption, psychological trauma and stressful life events and immune regulatory protein defects^5^,^6^. However, for half of the patients with RSA, a definitive cause for recurrent miscarriages cannot be identified^7^. Thus, it is urgent that the factors involved in RSA are identified and that we gain a clearer understanding of its causes.

The placenta, a maternal-fetal barrier, provides the developing fetus with all of the nutrients necessary for its development^8^. To effectively exchange nutrients and waste between mother and fetus throughout gestation, the placenta needs to maintain its own circulation and metabolism via angiogenesis^9^. Angiogenesis involves the formation of new branches from pre-existing vessels and the remodeling of an existing vascular network^10^. The three steps of angiogenesis—initiation, proliferation-invasion, and maturation-differentiation—are all critical for normal uteroplacental development^11^. Abnormal angiogenesis is considered one of the most important causes of RSA. Identifying a possible mechanism for the regulation of placental angiogenesis might lead to a treatment to combat RSA.

miRNAs are a class of conservative, 19- to 24-nucleotide non-protein-coding RNAs that can regulate gene expression by targeting messenger RNAs (mRNAs) via binding to specific sites in the 3′UTR, resulting in translational repression, cleavage or destabilization^12^,^13^. miRNAs elicit critical changes in gene expression programs that underlie diverse aspects of biology, such as embryogenesis and regulation of cellular differentiation, metabolism, proliferation and apoptosis^14^. An increasing number of studies have demonstrated that miRNAs are expressed abundantly in the human placenta, and dysregulation of miRNAs has been associated with RSA pathogenesis^7^,^15^.

The objectives of the current study were to investigate the expression level of miR-16 in RSA, to evaluate the role of miR-16 on placental angiogenesis both *in vitro* and *in vivo*, to provide evidence for the regulation of VEGF expression by miR-16 and to reveal the role this regulation plays in the pathogenesis of RSA (see Supplementary Fig. S1).

## Results

### The abundance of mir-16 in the villi and deciduais elevated in RSA patients

**Figure 1:**
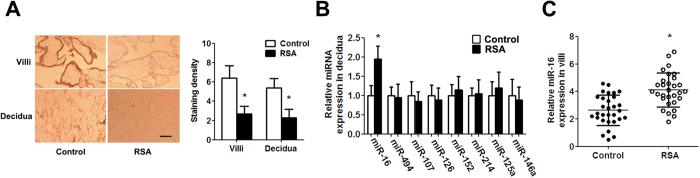
Aberrantly high miR-16 expression in RSA cases. (**A**) Expression of VEGF in the villi and decidua as revealed by immunohistochemistry (scale bar = 50 μm). The immunostaining intensity of VEGF was evaluated according to IRS. (**B**) Mature miRNA expression was determined by TaqMan qPCR in the decidua. (**C**) miR-16 expression was determined by TaqMan qPCR in villi. Data are expressed as the mean ± SD. *P < 0.05 *vs.* control group, n = 30.

Immunohistochemistry revealed that VEGF expression was remarkably lower in RSA patients compared with control pregnant women (Fig. 1A). To investigate whether a specific miRNA regulates VEGF expression in RSA, we detected a total of 8 potential miRNAs that targeted the VEGF gene. The expression of these 8 miRNAs was demonstrated by qPCR. We found that the expression of miR-16 in the decidua of the RSA group was significantly greater than that in the decidua of the control group (Fig. 1B). The higher expression of miR-16 was confirmed in the villi of RSA cases (Fig. 1C). Therefore, these results suggested that miR-16 is overexpressed in RSA.

### mir-16 inhibits placentation and angiogenesis *in vitro* and *in vivo*

**Figure 2:**
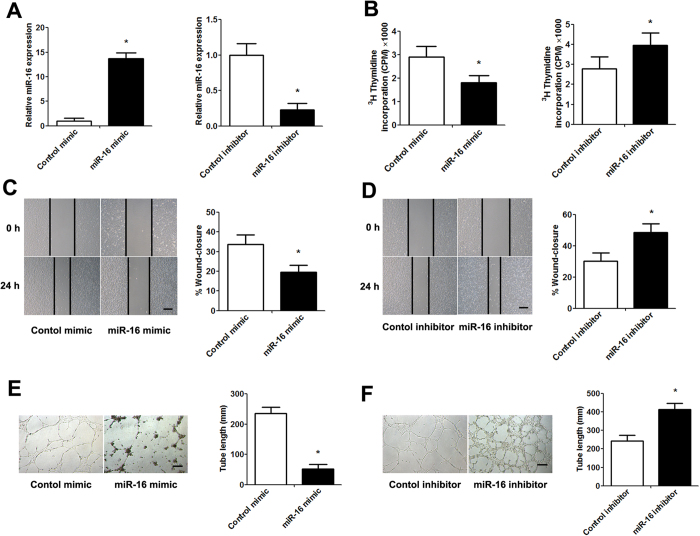
The effect of miR-16 on HUVEC proliferation, migration and tube formation. HUVECs were transfected with control or miR-16 mimic/inhibitor for 48 h. (**A**) qPCR analysis was performed to examine miR-16 levels. (**B**) The level of ^3^H-thymidine incorporation was measured after transfection. (**C**,**D**) Representative images were taken at 0 and 24 h to assess the cell migration into the open space (scale bar = 50 μm). Quantification of the percentage of the distance migrated was achieved by measuring these distances in 5 high-power fields. (**E**,**F**) Representative images of tube formation (scale bar = 50 μm). Quantification of the vascular tube structure was conducted by measuring the total tubule length in 5 high-power fields. Data are expressed as the mean ± SD. *P < 0.05 *vs.* control mimic/inhibitor group, n = 5.

Considering the inhibitory effect of miR-16 on VEGF expression, which is related to placental angiogenesis, maintenance and stabilization, we assessed the anti-angiogenic activity of miR-16 by transfecting Human Umbilical Vein Endothelial Cells (HUVECs) with a miR-16 mimic/inhibitor *in vitro*. qPCR analysis demonstrated that transfecting HUVECs with the miR-16 mimic increased the miR-16 levels by approximately 14-fold over the endogenous transcription level. In contrast, transfecting HUVECs with a miR-16 inhibitor significantly decreased the miR-16 levels (Fig. 2A). It is known that angiogenesis demands the proliferation and migration of endothelial cells. The effects of miR-16 on the VEGF-induced proliferation and migration of HUVECs were evaluated. The overexpression of miR-16 in HUVECs significantly inhibited HUVEC proliferation and migration, whereas the reduction of miR-16 expression promoted HUVEC proliferation and migration (Fig. 2B–D). In tube formation assays, the length of formed tubules was calculated using inverted phase contrast microscopy, which directly revealed the ability of the HUVECs to form tubular structures. Moreover, we discovered that miR-16 overexpression significantly repressed HUVEC tube formation, whereas transfection with the miR-16 inhibitor increased tube formation (Fig. 2E,F).

**Figure 3:**
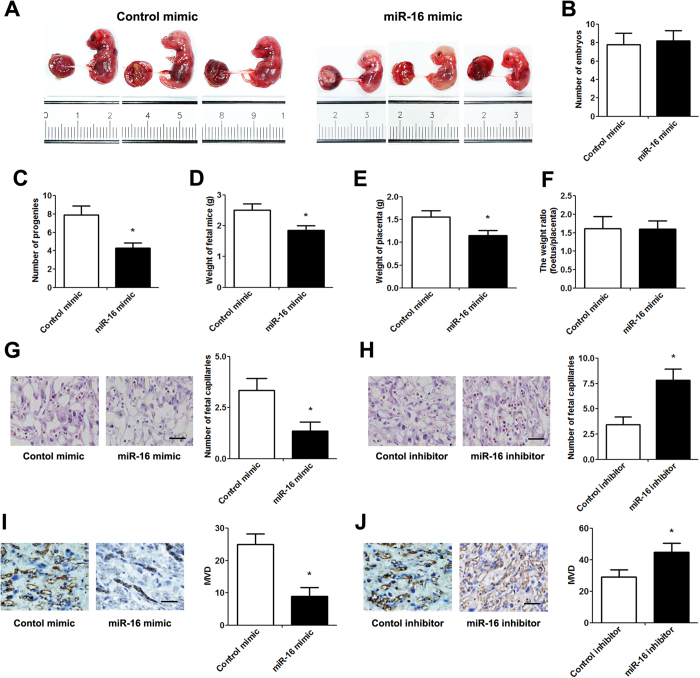
Impact of miR-16 on placentation and angiogenesis. Cholesterol-conjugated miRNAs (10 nmol) were locally injected into the placenta using apercutaneous ultrasound-guided approach at 7.5-days of embryogenesis. (**A**) Mouse fetuses and placentas at 14.5days of embryogenesis. (**B**) The number of embryos at pregnancy. (**C**) Average litter size after birth. (**D**) The weight of fetal mice.(**E**) The weight of placenta. (**F**) The weight ratio (fetus/placenta). (**G,H**) Histological analysis of decidua from different groups using hematoxylin and eosin staining (scale bar = 50 μm). The capillaries were manually counted in 5 high-power fields. (**I,J**) CD34 staining by immunochemistry in decidua (scale bar = 50 μm). MVD was assessedusingthe Weidner method. Data are expressed as the mean ± SD. *P < 0.05 *vs.* control mimic/inhibitor group, n = 5.

To identify the effect of miR-16 on placental angiogenesis *in vivo*, cholesterol-conjugated miRNAs were locally injected into the placenta using a percutaneous ultrasound-guided approach on day 7.5 of pregnancy. Mice fetuses and placentas were collected 7 days after injection (Fig. 3A). Although miR-16 overexpression did not affect the number of embryos, the number of progeny was significantly reduced after miR-16 injection (Fig. 3B,C). Moreover, the weights of the placenta and embryo were significantly decreased in the miR-16 overexpression group compared with the control group (Fig. 3D,E). However, the weight ratio of fetus/placenta at 14.5 days of embryogenesis did not differ between the two groups (Fig. 3F). Subsequent histologic analysis revealed that the total placental vasculature and the number of capillaries were significantly inhibited by miR-16 mimic injection but significantly promoted by miR-16 inhibitor injection (Fig. 3G,H, Table 1). Immunohistochemistry also showed similar results in which miR-16 overexpression inhibited placental angiogenesis, whereas the inhibition of miR-16 expression induced placental angiogenesis (Fig. 3I,J).

### VEGF is a direct target of mir-16

**Figure 4:**
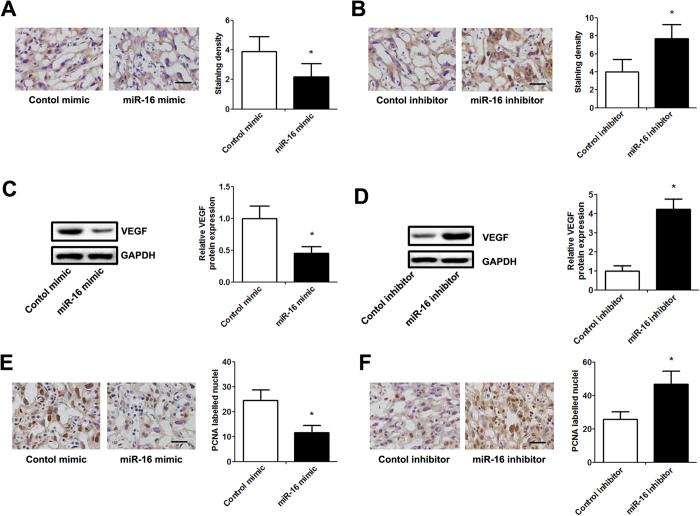
miR-16 regulates VEGF expression and placental proliferation *in vivo*. Cholesterol-conjugated miRNAs (10 nmol) were locally injected into the placentas of pregnant mice for 7 days. (**A**,**B**) Representative images of immunostaining for VEGF in decidua from different groups (scale bar = 50 μm). The immunostaining intensity was evaluated according to IRS. (**C**,**D**) The expression of VEGF protein was analyzed by western blot. The relative amount of VEGF was normalized to the internal protein GAPDH. (**E**,**F**) Representative images of immunolabeling for PCNA in decidua from different groups (scale bar = 50 μm). The intensity of PCNA expression was evaluated in a manner corresponding to positively labelled nuclei in 5 high-power fields. Data are expressed as the mean ± SD. *P < 0.05 *vs.* control mimic/inhibitor group, n = 5.

miRNAs exert their function mainly by targeting the 3′UTR of target mRNAs. To demonstrate whether miR-16 affects the expression of VEGF, we first evaluated the correlation between VEGF expression and the miR-16 level in placentas of pregnant mice. Immunohistochemistry and western blotting confirmed that the VEGF protein expression level was significantly decreased by miR-16 mimic injection but significantly elevated by miR-16 inhibitor injection (Fig. 4A–D). In addition, overexpression due to injection of the placenta with miR-16 mimics significantly decreased the protein expression of Proliferating Cell Nuclear Antigen (PCNA). However, injection with miR-16 inhibitors significantly upregulated the PCNA protein levels (Fig. 4E,F).

**Figure 5:**
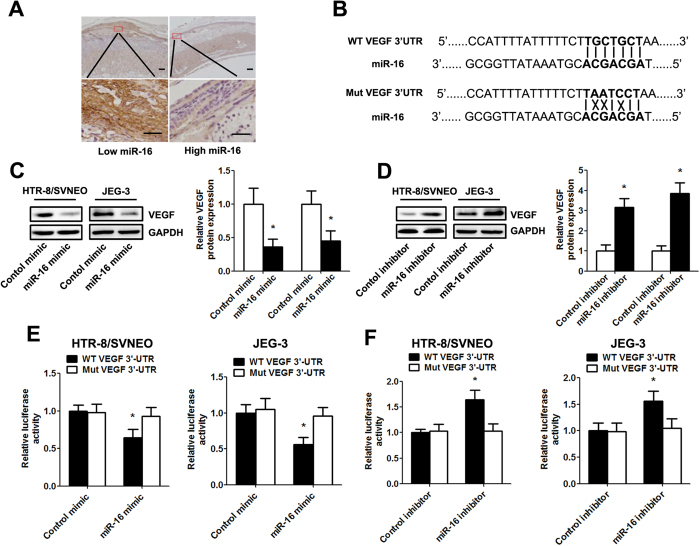
miR-16 directly targeted VEGF 3′UTR and down-regulates its expression. (**A**) An inverse relationship between the expression of miR-16 and VEGF was found in clinical decidual specimens of RSA patients (scale bar = 50 μm). (**B**)Predicted miR-16 target sequences in the 3′UTR of VEGF are shown (solid lines indicate matching base pairs and crosses represent non-matching base pairs). (**C**,**D**) Western blotting analysis of HTR-8/SVNEO and JEG-3 cells following miRNA mimic/inhibitor transfection for 48 h. (**E**,**F**) HTR-8/SVNEO and JEG-3 cells were co-transfected with the luciferase reporter plasmid carrying the wild type/mutant binding sequences of VEGF and the miRNA mimic/inhibitor. A dual-luciferase reporter system analysis was performed. Data are expressed as the mean ± SD. *P < 0.05 *vs.* control mimic/inhibitor group, n = 5.

In the clinical specimens, the VEGF levels in the decidua with high miR-16 expression were significantly lower than those in the decidua with low miR-16 expression (Fig. 5A). To verify whether miR-16 directly suppresses VEGF expression, we analyzed the potential miR-16 seed sequence in the 3′UTR of VEGF using the online publically available algorithms (TargetScan andmiRanda) and cloned a wild-type or mutant fragment containing the binding sequence (376 bp) into the luciferase reporter gene system (Fig. 5B). Western blot analysis revealed that VEGF expression was significantly downregulated by miR-16 mimic transfection in HTR-8/SVNEO and JEG-3 cells. However, the inhibition of miR-16 upregulated VEGF levels (Fig. 5C,D). The miR-16 mimic/inhibitor and the redesigned luciferase reporter plasmid were then co-transfected into HTR-8/SVNEO and JEG-3 cells. The results showed that overexpression of miR-16 in both cell lines led to significantly reduced luciferase activity for the wild-type, whereas the miR-16 knockdown increased wild-type luciferase activity. In contrast, the activity of the luciferase reporter gene linked to the 3′UTR of mutant VEGF did not change in the presence of the miR-16 mimic/inhibitor (Fig. 5E,F).

## Discussion

The identification and validation of novel biomarkers for RSA is a high priority not only for the diagnosis and clinical follow-up of RSA but also for defining novel therapeutic strategies. During the past few years, although there have been substantial advances in the awareness of RSA, major limitations still exist in managing RSA. Endometrial angiogenesis and decidualization are prerequisites for a successful implantation and a good outcome of pregnancy. In the uterus during pregnancy, critical angiogenic signals likely are produced by the decidualizing endometrial cells acting on the endothelial cells to promote their proliferation, migration and differentiation^16^. Accumulating evidence shows that placental angiodysplasia and endothelial dysfunction may contribute to cases of RSA^17^,^18^. A majority of pregnancies that result in miscarriage are believed to be caused by defective placentation associated with an absence of physiological changes in maternal spiral arteries^19^.

miRNAs are believed to play important roles in a variety of gynecological reproductive diseases including leiomyoma, endometriosis, and RSA^20^. Indeed, there is a differential expression of miRNAs in the villi and decidua of RSA patients, and all significantly differentially expressed miRNAs affect pregnancy by regulating adhesion. Furthermore, a few of the miRNAs can lead to RSA by regulating the expression of angiogenesis-related genes. All of these miRNAs act together to regulate a variety of physiological functions, leading to RSA. One study suggested that miR-16 inhibits angiogenesis, trophoblast cell proliferation, and migration, and inhibits angiogenesis through VEGF suppression^21^. These data imply that miR-16 may play a critical role in placental angiogenesis.

In the present study, we initially detected the expression level of miR-16 in samples of RSA patients and control women. Importantly, quantification of the data indicated that miR-16 was expressed at significantly higher levels in the villi and decidua of the RSA patients than that in the controls. These results indicate that the status of miR-16 expression should be determined in RSA patients. *In vitro*, miR-16 overexpression in HUVECs suppresses proliferation, migration and tube formation, suggesting that miR-16 may be a novel suppressor of and may play a critical role in angiogenesis. Moreover, injection with miR-16 in the placenta of pregnant mice caused remarkably decreased placental vasculature and microvascular density, leading to aberrant placentation and spontaneous abortion. A recent study indicated that miR-16 inhibits the proliferation, migration and angiogenesis-regulating potential of mesenchymal stem cells^22^. Our data are consistent with a previous study demonstrating that miR-16 exerts inhibitory biological features during placental angiogenesis.

In addition, we validated VEGF as a direct target gene for miR-16, and we found that miR-16 negatively regulates VEGF expression by directly targeting the 3′UTR of VEGF mRNA in HTR-8/SVNEO and JEG-3 cells. VEGF, acting as a key angiogenesis promoter, plays a significant role in physiological and pathophysiological vascular development and maintenance^23^. It has been shown that VEGF expression is found in the decidua cells of early pregnancy, and it participates in physiological placental angiogenesis during embryogenesis and reproductive functions during the initial stages of pregnancy^24^. Dysregulation of VEGF expression has been linked to the onset of placental pathologies including preeclampsia, early pregnancy loss and intrauterine growth restriction and is deeply implicated in multiple steps of RSA occurrence and development^25^,^26^. Previous studies have indicated that early pregnancy loss is associated with low VEGF expression and that altered VEGF expression is likely to contribute to the etiology of RSA^27^,^28^. In this study, we demonstrated that miR-16 mediates the reduction of VEGF and found that normal VEGF expression is a notable feature and may be one of the many critical events that occur in placental angiogenesis. Aberrant angiogenesis may be partially attributed to overexpressed miR-16 in the placenta of RSA patients.

Overall, the current study found that miR-16 expression is upregulated in the villi and decidua of RSA patients. Our data revealed that the important molecular mechanism by which miR-16 exerts its negative effects on placental angiogenesis occurs via VEGF suppression. However, our work is merely a starting point for the study of miRNAs in RSA, and the exact regulatory mechanism and related signaling pathways of miR-16in RSA still require more experimental evidence to verify. Taken together, the identification of miR-16 provides a potential diagnostic marker and therapeutic target for RSA patients.

## Materials and Methods

### Patients and Tissue Samples

A prospective case-control study was conducted between October 2013 and November 2014 in the Key Laboratory of Fertility Preservation and Maintenance of Ministry of Education, Yinchuan, China. A total of 30 women aged 25–31 years with a history of three or more spontaneous abortions and 30 fertile women aged 24–31 years with one or more children and no history of spontaneous abortions were enrolled in this study between 2013 and 2014 in the Department of Obstetrics and Gynecology, General Hospital of Ningxia Medical University. Baseline characteristics of all women were recorded (Table 2). Placental villi and decidua tissue were also obtained from cases of induced abortion between 6 and 12 gestational weeks. Medical abortion was performed as previously described^29^. All samples were examined to exclude chromosomal, anatomical, hormonal and infectious pathologies. All tissue samples were snap-frozen in liquid nitrogen for real-time polymerase chain reaction (qPCR) analyses or were fixed in 10% formalin and embedded in paraffin for immunohistochemical analyses. The institutional ethics committee of the Ningxia Medical University approved the study protocol, and written informed consent was obtained from participants. The human experiments were performed in accordance with the relevant guidelines, including any relevant details.

### miRNA expression assay

Total RNAs were extracted using TRIzol reagent (Invitrogen, Carlsbad, CA, USA) according to the manufacturer’s instructions. cDNA was synthesized from total mRNA (5 μg) with specific reverse transcription primers. The expression of miRNAs was quantified using a miRNA-specific TaqMan miRNA Assay Kit (Applied Biosystems, Foster City, CA, USA). qPCR was performed with a FastStart Universal SYBR Green Master (Roche Diagnostics, Mannheim, Germany). Quantitative PCR reactions utilized TaqMan Universal PCR Master Mix and TaqMan miRNA Assays (ABI, Foster City, CA, USA) according to the manufacturer’s directions. The relative quantification of miRNAs was calculated using the 2^−△△^Ct method. The miRNA levels were normalized using U6 small nuclear RNA as an internal control and measured relative to a calibrator sample as the external control.

### Cell Culture and Transfection

The choriocarcinoma cell line JEG-3 and the immortalized human trophoblast cell line HTR-8/SVNEO were obtained from the American Type Culture Collection and grown in RPMI-1640 medium (Gibco, Carlsbad, CA, USA) supplemented with 10% fetal bovine serum (FBS, Hyclone, Logan, UT, USA) and 100 U/ml penicillin/streptomycin (Invitrogen, Carlsbad, CA, USA) in humidified air at 37 °C and 5% CO_2_. The miR-16 mimics and control mimics were purchased from Ambion (Austin, TX, USA). For miRNA transfection, cells were plated in 6-well plates and cultured to 50–70% confluence prior to transfection of the mimic or inhibitor using Lipofectamine2000 (Invitrogen, Carlsbad, CA, USA) according to the manufacturer’s instructions. All further analyses were performed 48 hours after transfection.

### ^3^H-thymidine incorporation assay

For the ^3^H-thymidine incorporation assays, HUVECs (1 × 10^5^ cells/well) were plated in 24-well plates and cultured until 60% confluence. Cells were pretreated with VEGF (Peprotech, Offenbach, Germany) for 24 h and serum-starved overnight, followed by the addition of serum containing ^3^H-thymidine (2 Ci/mM) for 6 h. The cells were then fixed in 0.3 ml of 10% trichloroacetic acid (TCA) and lysed in 100 μl of 0.2 M NaOH/0.2% SDS. ^3^H-thymidine incorporation was measured by scintillation counting in a Packard Scintillation Analyzer (Covina, USA).

### Migration assay

For the migration assay, monolayer HUVECs were wounded by scratching the plate with a sterile 200-μl pipette tip. The cells were then washed several times with PBS to remove cell debris, and the culture was continued in fresh medium. Images of the cells were obtained with an inverted phase contrast microscope (Olympus, Tokyo, Japan) at 0 h and 24 h after scratching. The migration capacity was calculated as the percentage of wound closure with the initial wound width defined as 100%.

### Tube formation assay

The formation of HUVECs into capillary-like structures was conducted as described previously^30^. At least 30 min before the experiment, 96-well plates were coated with Matrigel (BD, Biosciences, Bedford, MA, USA). HUVECs (5 × 10^3^ cells/well) were cultured in Ham’s F12K basal medium (Gibco, Carlsbad, CA, USA) with 200 ml tumor cell-conditioned medium. Tumor cell-conditioned medium was prepared from 1 × 10^6^ JEG-3 cells transfected with a miR-16 mimic or inhibitor, which were cultured in the same conditions for 48 h. Images showing the formation of capillary-like structures were obtained after 12 h with an inverted microscope (Olympus, Tokyo, Japan) at 50 ×  magnification. Tubular structures were assessed by measuring the total tubule length in each field of view using Image-Pro Plus software.

### Animals

Wild-type C57BL/6 mice (8 to 12 weeks old) were maintained on an autoclaved chow diet in filter-topped cages in a specific pathogen-free (SPF) animal room (controlled temperature (24 ± 2 °C), with approximately 40% humidity and a 12/12 h light/dark cycle. The presence of a vaginal plug was designated as day 0.5 of pregnancy. To deliver cholesterol-conjugated miRNAs (Austin, TX, USA), 10 nmol miRNAs in 50 μl saline buffer was locally injected into the placenta using apercutaneous ultrasound-guided approach on day 7.5 of pregnancy. All placentas in a single litter received the same miRNA (control or miR-16 mimic/inhibitor) injection. Pregnant females were killed on day 14.5 of pregnancy, and placenta samples were collected. All animal experiments were approved by the Ethics Review Board of the Ningxia Medical University and conducted following institutional guidelines.

### Immunohistochemistry assay

Paraffin-embedded tissue sections (4-μm thick) were deparaffinized in xylene and rehydrated through a graded series of methanol. Endogenous peroxidase activity was quenched with 3% H_2_O_2_ for 10 min. Subsequently, the slides were subjected to antigen retrieval in a microwave oven in Tris-buffered citric acid (pH = 6) for 10 min. The sections were incubated in 10% goat serum albumin in phosphate buffered saline (PBS) for 30 min, followed by incubation with a mouse monoclonal anti-VEGF antibody (C-1, 1/50 dilution, Santa Cruz Biotechnology, Santa Cruz, CA, USA), rabbit polyclonal anti-PCNA antibody (1/500 dilution; Abcam, Cambridge, UK) or rabbit monoclonal anti-CD34 antibody (1/50 dilution, Boshide Biotechnology Company, Wuhan, China) at 4 °C overnight. The sections were then incubated with the appropriate secondary antibody for 1 h and washed with PBS. Lastly, the visualization signal was developed by incubating the sections in 3, 3′-diaminobenzidine in buffered substrate for 5 min.

The immunostaining intensity was evaluated according to the immunoreactive score (IRS). IRS = staining intensity × percentage of positive cells. Staining intensity was scored as 0 (negative), 1 (weak), 2 (moderate), or 3 (strong). The percentages of positive cells were scored as 0 (0% positive cells), 1 (<10% positive cells), 2 (10–50% positive cells), 3 (51–80% positive cells), or 4 (>80% positive cells). The microvascular density (MVD) was determined using the Weidner method on the basis of CD34 staining^31^.

### Western blot assays

Protein extraction and western blotting were performed as previously described in more detail^32^. Briefly, proteins (50 μg) were separated by 10% sodium dodecyl sulphate-polyacrylamide gel (SDS-PAGE) and transferred to a nitrocellulose membrane (Amersham Bioscience, Buckinghamshire, U.K.). Membranes were blotted with an anti-VEGF antibody (sc-152, 1/1000 dilution, Santa Cruz Biotechnology, Santa Cruz, CA) overnight at 4 °C and then incubated with horseradish peroxidase-conjugated secondary antibody (Transduction Laboratories, Lexington, UK). GAPDH antibody (1/1000 dilution, Cell Signaling Technology, Boston, MA, USA) was used as a control. The bands were detected with Enhanced Chemiluminescence (Amersham Biosciences, Piscataway, NJ) and visualized with the ChemiDoc XRS System (Bio-Rad, Hercules, CA, USA).

### Plasmid construction

To generate a luciferase reporter vector, a 376-bp fragment of the VEGF 3′UTR sequence, as well as the mutant VEGF sequence, were synthesized by PCR. The primers contained the following restriction sites: VEGF 3′UTR forward, 5′-GGAATTCCCACACCATCACCATCGACAGA-3′, reverse, 5′-CAAGCTTGACACCAATAACATTAGCACTG-3′; and Mut VEGF 3′UTR forward, 5′-TTATTTTTCTTAATCCTAAATCACCGA-3′, reverse, 5′- TCGGTGATTTAGGATTAAGAAAAATAA-3′. The PCR product was cloned into the EcoRΙ and HindΙΙΙ restriction sites downstream of the luciferase open reading frame in the pGL3-luciferase reporter plasmid (Promega, Madison, WI, USA). The correct clones were confirmed by sequencing.

### Luciferase reporter assays

The cells (HTR-8/SVNEO and JEG-3) were grown to 70–80% confluence in 24-well plates and co-transfected with the luciferase reporter vector described above (200 ng) and the appropriate miRNA (50 nM) using Lipofectamine^TM^ 2000 reagent (Invitrogen, Carlsbad, CA, USA) according to the manufacturer’s instructions. After 48 h, cells were washed and lysed with passive lysis buffer (Promega, Madison, WI, USA), and luciferase activity was analyzed using dual luciferase assays (Promega, Madison, WI, USA). The relative reporter activity was obtained by normalization to the Renilla luciferase activity.

### Statistical analysis

SPSS13.0 software (SPSS Inc., Chicago, IL, USA) was used for statistical analyses. Values are expressed as the mean ± standard deviation (SD). Significant differences between groups were analyzed using Student’s t-test. Differences were considered statistically significant at P < 0.05.

## Additional Information

**How to cite this article**: Zhu, Y. *et al.* MicroRNA-16 inhibits foeto-maternal angiogenesis and causes recurrent spontaneous abortion by targeting vascular endothelial growth factor. *Sci. Rep.* **6**, 35536; doi: 10.1038/srep35536 (2016).

## Supplementary Material

Supplementary Information

## Figures and Tables

**Table 1:** Comparison of placental morphological parameters.

Morphological parameters	Control mimic (n = 5)	miR-16 mimic (n = 5)	Control inhibitor (n = 5)	miR-16 inhibitor (n = 5)
Proportion of spongiotrophoblast	0.206 ± 0.047	0.208 ± 0.021	0.205 ± 0.026	0.207 ± 0.028
Proportion of labyrinth	0.788 ± 0.057	0.791 ± 0.066	0.781 ± 0.049	0.790 ± 0.061
Total volume of spongiotrophoblast (mm^3^)	249 ± 78.6	148 ± 43.4*	254 ± 68.8	343 ± 72.6^#^
Total volume of labyrinth (mm^3^)	609 ± 102	449 ± 78.1*	599 ± 108	849 ± 126^#^
Total volume of fetal capillaries (mm^3^)	72.3 ± 15.6	41.6 ± 10.2*	70.8 ± 14.3	102.7 ± 18.8^#^
Exchange barrier (μm)	2.02 ± 0.056	5.13 ± 0.176*	1.98 ± 0.069	1.06 ± 0.044^#^

*P < 0.05 *vs.* control mimic; ^#^P < 0.05 *vs.* control inhibitor.

**Table 2:** Clinical characteristics of subjects.

Characteristics	Control (n = 30)	RSA (n = 30)	*P* value
Age (years)	30.3 ± 1.8	30.8 ± 2.2	NS
Gestational age (weeks)	11.9 ± 2.1	12.6 ± 2.6	NS
Smoking	none	none	NS
Pregnancy time (weeks)	10.8 ± 1.2	11.2 ± 1.9	NS
Median maternal weight (kg)	52.8 ± 4.5	53.4 ± 5.3	NS
Median maternal BMI (kg/m^2^)	22.3 ± 2.5	21.6 ± 1.9	NS
