# Supplementary material for: MicroRNA-16 inhibits feto-maternal angiogenesis and causes recurrent spontaneous abortion by targeting vascular endothelial growth factor
Source: Sci Rep. 2016 Oct 17;6:35536. doi: 10.1038/srep35536 (PMC5066269; doi:10.1038/srep35536)
Supplement: Supplementary Information [file srep35536-s1.pdf]

# MicroRNA-16 inhibits foeto-maternal angiogenesis and causes recurrent spontaneous abortion by targeting vascular endothelial growth factor

Yongsheng Zhu[1,#], Hong Lu[2,#], Zhenghao Huo[2,*], Zhanbin Ma[2], Jie Dang[2], Wei Dang[3], Lin Pan[4], Jing Chen[2], Huijun Zhong[2]

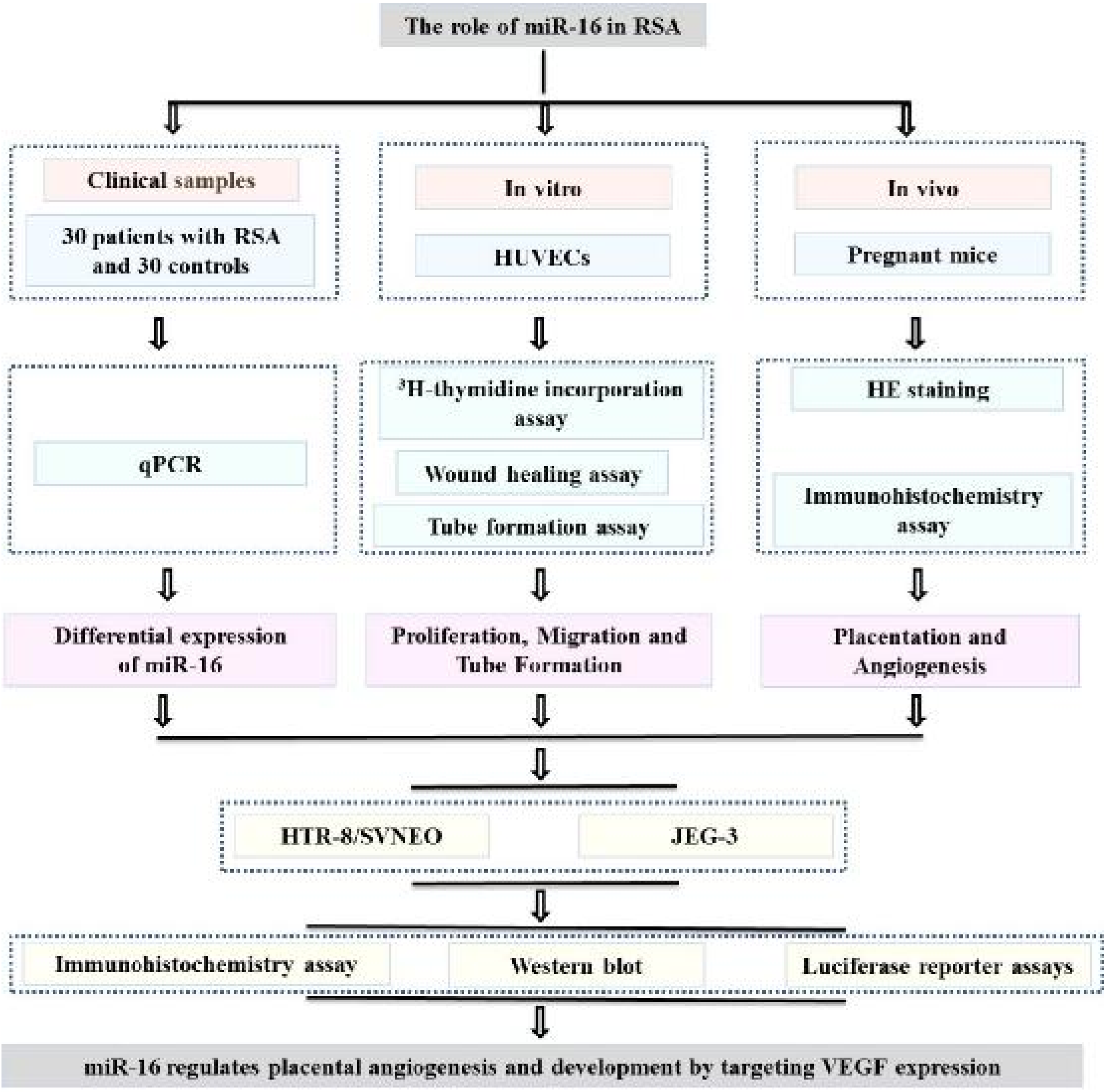


**Supplementary Figure S1** Model illustrating that technical route in this work.
